# Choroidal Thickness in Relation to Bone Mineral Density with Swept-Source Optical Coherence Tomography

**DOI:** 10.1155/2021/9995546

**Published:** 2021-09-25

**Authors:** Lin Jiang, Yiwen Qian, Qingjian Li, Jing Jiang, Yu Zhang, Xin Che, Zhiliang Wang

**Affiliations:** ^1^Health Management Center, Huashan Hospital of Fudan University, Shanghai, China; ^2^Department of Ophthalmology, Huashan Hospital of Fudan University, Shanghai, China; ^3^Department of Ophthalmology, Xiang'an Hospital of Xiamen University, Fujian Provincial Key Laboratory of Ophthalmology and Visual Science, Eye Institute of Xiamen University, School of Medicine, Xiamen University, Xiamen, Fujian, China

## Abstract

**Purpose:**

To assess whether bone mineral density, indicated by the lumbar X-ray scan, is related to changes in choroid thickness in normal subjects.

**Methods:**

This study included 355 patients with decreased bone mineral density and 355 age- and sex-matched healthy subjects. Lumbar BMD was measured by dual-energy X-ray absorptiometry (DXA). Choroidal thickness was measured using swept-source optical coherence tomography (SS-OCT). Blood pressure (BP), cholesterol, triglyceride (TG), high-density lipoprotein (HDL), and low-density lipoprotein (LDL) were recorded on the same day.

**Results:**

There was a significant difference in average choroidal thickness between low BMD subjects and normal subjects (*p*=0.003). The BP, cholesterol, triglyceride, HDL, and LDL showed no significant difference between the two groups. The correlations showed that average choroidal thicknesses were associated with BMD in the entire population (*r* = 0.125, *p*=0.001).

**Conclusion:**

The choroidal thickness is thinner in low BMD populations compared with normal individuals. There is a strong positive correlation of choroidal thickness with BMD, regardless of age, sex, and other demographic and clinical factors.

## 1. Introduction

Osteoporosis is a polygenic, degenerative disease correlated with various systemic diseases [1]. Vascularization is fundamental for bone formation and bone tissue homeostasis. Patients with bone loss are prone to develop various vascular diseases, such as atherosclerosis and arterial stiffness [2–4]. And bone loss is also associated with various systemic immune and inflammatory diseases, such as Graves' disease [5] and systemic lupus erythematosus (SLE) [6, 7].

In addition, osteoporosis is also linked to eye diseases, such as age-related macular degeneration (AMD). There are numerous studies in the literature which have shown that low bone mineral density (BMD) was significantly associated with the progression of AMD [8], while higher levels of BMD may be associated with a lower risk for AMD [9]. However, the mechanism is still intriguing. AMD is the most common progressive outer retina disorder with the pathogenesis of the choroidal vasculature, oxidative stress, and inflammation [10, 11]. It is believed that hypoperfusion of the choroidal vasculature could lead to AMD progression [12].

The choroid, a dense vascular plexus of the eye, is composed of melanocytes, fibroblasts, immune cells, collagen fibers, and elastic connective tissue. The blood vessel of the choroid is of vital importance in nourishing the outer segment of the retina and the retinal pigment epithelium (RPE). The unique features of the choroid may render choroidal microvessels more prone to undergo structural changes if the system is stressed [12].

To the best of our knowledge, there have been no studies evaluating subfoveal choroidal thickness with osteoporosis. The study aims to evaluate the relationship between the BMD and choroidal vasculature in normal individuals.

## 2. Materials and Methods

### 2.1. Subjects

The study included a total of 2732 participants who were referred for BMD analysis at Huashan Hospital from March 2019 to October 2019. The study was approved by the institutional ethics committee of Huashan Hospital affiliated to Fudan University and conducted in accordance with the tenets of the Declaration of Helsinki. 355 eligible individuals with low BMD and 1 : 1 age- and gender-matched subjects were enrolled in this study. The mean age of the participants was 54.41 y in the low BMD group and 54.98 y in the control group separately (range: from 30 y to 75 y). They underwent SS-OCT examination to detect the choroidal thickness. Body mass index (BMI) was calculated based on body weight and height. The systolic and diastolic blood pressures (BPs) were measured with a mercury sphygmomanometer at rest, and the mean arterial pressure (MAP) was calculated as diastolic BP +1/3 (systolic BP − diastolic BP). Fasting blood samples including total cholesterol, triglyceride (TG), high-density lipoprotein (HDL), and low-density lipoprotein (LDL) were taken on the same day for analysis. All study participants had best-corrected visual acuity (BCVA) of 20/25 or more, with a refractive error in the range +3.0 to −3.0 diopters and intraocular pressure (IOP) between 10 mmHg and 21 mmHg. Those with systemic diseases such as hypertension, diabetic mellitus, cardiovascular disease, and renal impairment were excluded. Patients on anticoagulation and antiosteoporotic agents except calcium and vitamin D were also not eligible. Patients with ocular diseases such as glaucoma, uveitis, retinal detachment, and a history of ophthalmic surgery that may have affected the choroidal vascular network were also excluded.

### 2.2. Swept-Source Optical Coherence Tomography Imaging

**Figure 1:**
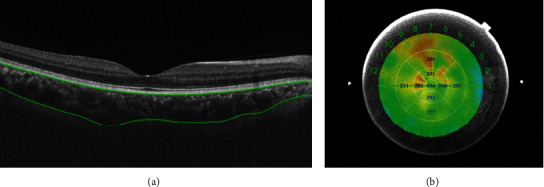
Representative images of choroidal thickness. (a) Choroidal thickness (between two green lines). (b) Choroidal thickness of nine sectors of the ETDRS grid.

OCT images were obtained with a SS-OCT (DRI OCT-1 Atlantis, version 9.31, Topcon Co., Tokyo, Japan), which overcomes the scattering of light on the choroid due to a longer wavelength of approximately 1050 nm. We obtained the choroidal thickness using in-built OCT software. The choroidal thickness was defined as the distance from the outer border of the RPE to the chorioscleral interface (CSI). Thickness maps in accordance with the standard Early Treatment Diabetic Retinopathy Study (ETDRS) subfield were created automatically which consist of 3 concentric circular areas (1 mm, 3 mm, and 6 mm separately) with nine independent sectors for analysis (Figure 1). For every OCT scan, the nine parts' choroidal thickness and the average one were measured automatically according to the standard ETDRS grid, and each segmented layer line can be manually adjusted to avoid possible errors. Only the right eye of each participant was assessed for statistical analysis. All measurements were performed during the same daily interval (8–10 am) to avoid diurnal variations in choroidal thickness and performed by the same skilled technician.

### 2.3. Measurement of Bone Mineral Density

BMD was assessed by a dual-energy X-ray absorptiometry (DXA) scanner that provides a rapid image with low radiation exposure. In our subjects, DXA was performed at the central lumbar spine (L2–4), and the density was defined as the mean value of the CT unit of measurement (milligrams per cubic centimeter, mg/cm^3^). Current criteria of low BMD were defined as a condition of *T*-score <1.5 or BMD minus 1.5 SD below the adult mean.

### 2.4. Statistical Analysis

Values were expressed as mean ± standard deviation. Continuous data were compared using Student's *t*-test. Pearson's correlation coefficient was used to test the relationships between choroidal thickness and BMD. Statistical signiﬁcance was deﬁned as 2-tailed *p* value <0.05. All statistical analyses were performed using SPSS Statistics version.

## 3. Results

Table 1 summarizes the completed demographic characteristics and clinical features of the overall study population (*n* = 710). 355 individuals with bone loss and 355 healthy controls were matched by sex and age (1 : 1 pairwise matching). The average BMD was 101.13 mg/cm^3^ for the low BMD group and 155.73 mg/cm^3^ for the healthy controls. No significant differences in age, sex, BMI, and BP were observed between the low BMD group and the control group. In addition, the two groups were also comparable regarding the blood analysis of total cholesterol, TG, HDL, and LDL.

**Figure 2:**
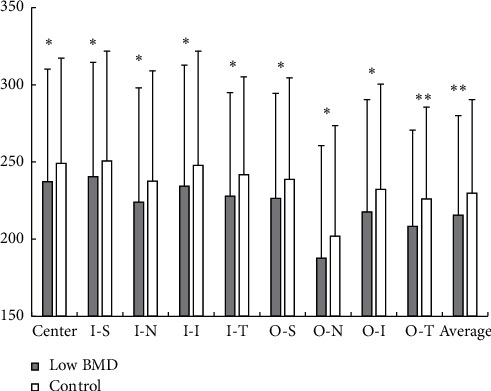
The choroidal thickness of nine sectors of the ETDRS grid in the low BMD compared with the controls. I-S: inner superior; I-N: inner nasal; I-I: inner inferior; I-T: inner temporal; O-S: outer superior; O-N: outer nasal; O-I: outer inferior; O-T: outer temporal.

Data showed that average choroidal thickness was significantly depressed in the low BMD than the control group (215.50 *μ*m vs. 229.73 *μ*m, *p*=0.003) (Table 2 and Figure 2). In our analysis, the choroidal thickness was thickest in the subfoveal and superior region while thinnest in the nasal and temporal region. Statistical analysis showed significance between the two groups for the choroidal thickness in all sectors except the inner superior sector. Among them, the inner temporal as well as the outer temporal location had the greatest difference (*p*=0.005 and *p* < 0.001 separately), while the inner superior showed no obvious difference (*p*=0.062) (Table 2 and Figure 2).

The correlations of choroidal thicknesses with the BMD level are shown in Table 3. Average choroidal thicknesses were associated with BMD in the entire population (*r* = 0.125, *p*=0.001).

## 4. Discussion

In the present study, the authors compared choroidal thickness in low BMD and healthy individuals to identify the association between bone loss and ocular vasculature. The results indicated a significant difference between bone loss and choroidal thinning in normal subjects. BMD is also affected by abnormal blood lipids [13, 14]. Therefore, we evaluated the blood lipids and found no significant difference between the low BMD group and the control group. The multivariate linear regression analysis revealed that BMD was independently associated with choroidal thickness.

Recently, different clinical studies have drawn the conclusion that low BMD is linked to the progression of AMD. A Korean cohort study showed that osteoporosis was associated with AMD in women while no association with other age-related eye diseases [8]. A multicenter study in the United States demonstrated that higher levels of BMD may be associated with a lower risk for AMD [9]. A prospective study demonstrated that vitamin D may prevent or delay the progression of AMD [15]. The relationship between the BMD and the ocular vasculature has never been studied.

The underlying physiology basis for the relationship between bone loss and choroidal thinning is intriguing. We concluded the following, may be the possible explanations. Firstly, low BMD may accelerate the deposition of minerals in the choroidal vasculature and cause local inﬂammation and oxidative stress which are thought to cause the hypoperfusion of the choroidal vasculature. Secondly, 25-hydroxyvitamin D (25(OH)D), which binds to the vitamin D receptor on the choriocapillaris, could protect the choroid from chronic inflammation and oxidative stress. Patients with low BMD tend to have a low level of vitamin D [16]. Furthermore, low BMD has a tendency of developing atherosclerosis and vascular stiffness, which might predispose the choroidal circulation to a greater hemodynamic change [3]. The hypoperfusion change will ultimately cause the choroidal vascular lesion and choroidal thinning.

In addition, vascular endothelial lesion is highly regulated and affected by mechanisms similar to those involved in bone mineralization, such as cathepsin K (CatK), osteoprotegerin (OPG), and osteopontin (OPN) [17]. Osteoporosis is believed to be closely related to endothelial dysfunction. Thus, vascular lesions anatomically contribute to the significant difference in choroidal thickness between the two groups.

Unlike retinal blood flow, which is autoregulated, choroidal blood flow is principally affected by the autonomous nervous system and circulatory hormones [18]. In the current analysis, the nasal and temporal sectors share the significant thinning of the choroid in low BMD individuals in accordance with other studies [19, 20], mainly because they are thinner than other sectors and more prone to be affected by systemic diseases.

The present study has several limitations. First, we lack the measure of atherosclerosis and vascular stiffness, which might suggest the relationship between the choroidal thickness and vascular abnormalities. Second, we did not assess the axial length, estrogen, and smoking amount of the individuals, which were believed to have a strong positive correlation with choroidal thickness. Third, we used choroidal thickness as the main target of choroidal vessels, while angio-OCT should be adopted to further confirm the correlation of the choroidal vasculature and bone loss.

Choroidal study is a relative new way which can help us in the understanding of the effects on systemic health on choroidal thickness. Our study demonstrated the possibility of the crosstalk between the bone loss and choroid, which might help new strategies for the prevention of choroid abnormalities.

## Figures and Tables

**Table 1:** Demographic and clinical features of the overall study population.

Parameter	Low BMD Mean (SD)	Controls Mean (SD)	*p* value
Patient, *n*	355	355	—
Eye, *n*	355	355	—
Gender, *n* (%)			
Male	210	210	
Female	145	145	
Age, years	54.41 (7.63)	54.98 (7.64)	0.321
Range	30–75	30–75	
Blood pressure			
SBP (mmHg)	119.72 (11.54)	120.22 (12.58)	0.582
DBP (mmHg)	73.80 (8.46)	74.50 (9.43)	0.301
MAP (mmHg)	89.10 (8.77)	89.74 (9.81)	0.367
BMI (kg/m^2^)	23.79 (2.85)	24.15 (3.04)	0.101
Cholesterol (mg/dL)	5.05 (1.07)	4.96 (0.94)	0.171
TG (mg/dL)	1.78 (1.44)	1.85 (2.03)	0.619
HDL (mg/dL)	1.31 (0.34)	1.28 (0.33)	0.299
LDL (mg/dL)	3.02 (0.90)	2.94 (0.78)	0.166

SBP: systolic blood pressure; DBP: diastolic blood pressure; BMI: body mass index; TG: triglyceride; HDL: high-density lipoprotein; LDL: low-density lipoprotein.

**Table 2:** The choroidal thickness of nine sectors of the ETDRS grid in the low BMD compared with the controls.

Choroidal thickness	Low BMD (*n* = 396) Mean (SD)	Control (*n* = 432) Mean (SD)	*p* value
Center, *μ*m	237.24 (72.87)	249.01 (68.23)	0.027
Inner superior, *μ*m	240.55 (73.98)	250.71 (71.08)	0.062
Inner nasal, *μ*m	223.99 (74.04)	237.63 (71.41)	0.013
Inner inferior, *μ*m	234.33 (78.35)	247.86 (73.93)	0.018
Inner temporal, *μ*m	227.87 (67.02)	241.77 (63.30)	0.005
Outer superior, *μ*m	226.37 (68.07)	238.66 (65.77)	0.015
Outer nasal, *μ*m	187.65 (72.96)	201.87 (71.72)	0.009
Outer inferior, *μ*m	217.67 (72.75)	232.15 (68.25)	0.006
Outer temporal, *μ*m	208.26 (62.38)	226.08 (59.41)	<0.001
Average thickness, *μ*m	215.50 (64.55)	229.73 (60.61)	0.003

**Table 3:** Correlation analysis results between choroidal thickness and BMD.

Choroidal thickness		Center	I-S	I-N	I-I	I-T	O-S	O-N	O-I	O-T	Average
BMD	*r*	0.077 ^*∗*^	0.063	0.092 ^*∗*^	0.096 ^*∗*^	0.112 ^*∗*^ ^*∗*^	0.096 ^*∗*^	0.090 ^*∗*^	0.133 ^*∗*^ ^*∗*^	0.181 ^*∗*^ ^*∗*^	0.125 ^*∗*^ ^*∗*^
	*p*	0.40	0.094	0.014	0.010	0.003	0.010	0.016	<0.001	<0.001	0.001

^*∗*^*p* < 0.05 and  ^*∗*^ ^*∗*^*p* < 0.01. I-S: inner superior; I-N: inner nasal; I-I: inner inferior; I-T: inner temporal; O-S: outer superior; O-N: outer nasal; O-I: outer inferior; O-T: outer temporal; BMD: bone mineral density.

## Data Availability

The data supporting the results of the study can be accessed by others in the manuscript.

## References

[1] Vassalle C., Mazzone A. (2016). Bone loss and vascular calcification: a bi-directional interplay?. *Vascular Pharmacology*.

[2] Choi S. H., An J. H., Lim S. (2009). Lower bone mineral density is associated with higher coronary calcification and coronary plaque burdens by multidetector row coronary computed tomography in pre- and postmenopausal women. *Clinical Endocrinology*.

[3] Van Campenhout A., Golledge J. (2009). Osteoprotegerin, vascular calcification and atherosclerosis. *Atherosclerosis*.

[4] Hofbauer L. C., Brueck C. C., Shanahan C. M., Schoppet M., Dobnig H. (2007). Vascular calcification and osteoporosis--from clinical observation towards molecular understanding. *Osteoporosis International*.

[5] Majima T., Komatsu Y., Doi K. (2006). Negative correlation between bone mineral density and TSH receptor antibodies in male patients with untreated Graves’ disease. *Osteoporosis International*.

[6] Mendoza-Pinto C., Rojas-Villarraga A., Molano-Gonzalez N. (2018). Bone mineral density and vertebral fractures in patients with systemic lupus erythematosus: a systematic review and meta-regression. *PloS One*.

[7] Guo Q., Fan P., Luo J. (2017). Assessment of bone mineral density and bone metabolism in young male adults recently diagnosed with systemic lupus erythematosus in China. *Lupus*.

[8] Yoo T. K., Kim S. H., Kwak J., Kim H. K., Rim T. H. (2018). Association between osteoporosis and age-related macular degeneration: the Korea national health and nutrition examination survey. *Investigative Opthalmology & Visual Science*.

[9] Seitzman R. L., Mangione C. M., Cauley J. A. (2007). Bone mineral density and age-related maculopathy in older women. *Journal of the American Geriatrics Society*.

[10] Kauppinen A., Paterno J. J., Blasiak J., Salminen A., Kaarniranta K. (2016). Inflammation and its role in age-related macular degeneration. *Cellular and Molecular Life Sciences*.

[11] Garcia-Layana A., Cabrera-López F., García-Arumí J., Arias-Barquet L., Ruiz-Moreno J. M. (2017). Early and intermediate age-related macular degeneration: update and clinical review. *Clinical Interventions in Aging*.

[12] van Lookeren Campagne M., LeCouter J., Yaspan B. L., Ye W. (2014). Mechanisms of age-related macular degeneration and therapeutic opportunities. *The Journal of Pathology*.

[13] Zhang Q., Zhou J., Wang Q. (2020). Association between bone mineral density and lipid profile in Chinese women. *Clinical Interventions in Aging*.

[14] Zheng J., Brion M. J., Kemp J. P. (2020). The effect of plasma lipids and lipid-lowering interventions on bone mineral density: a mendelian randomization study. *Journal of Bone and Mineral Research*.

[15] Merle B. M. J., Silver R. E., Rosner B., Seddon J. M. (2017). Associations between vitamin D intake and progression to incident advanced age-related macular degeneration. *Investigative Opthalmology & Visual Science*.

[16] Layana A. G., Minnella A. M., Garhöfer G. (2017). Vitamin D and age-related macular degeneration. *Nutrients*.

[17] Ye C., Xu M., Wang S. (2016). Decreased bone mineral density is an independent predictor for the development of atherosclerosis: a systematic review and meta-analysis. *PLoS One*.

[18] Nickla D. L., Wallman J. (2010). The multifunctional choroid. *Progress in Retinal and Eye Research*.

[19] Wang J., Jiang J., Zhang Y., Qian Y. W., Zhang J. F., Wang Z. L. (2019). Retinal and choroidal vascular changes in coronary heart disease: an optical coherence tomography angiography study. *Biomedical Optics Express*.

[20] Ahmad M., Kaszubski P. A., Cobbs L., Reynolds H., Smith R. T. (2017). Choroidal thickness in patients with coronary artery disease. *PloS One*.

